# Cloning and characterization of two Argonaute genes in wheat (*Triticum aestivum* L.)

**DOI:** 10.1186/1471-2229-13-18

**Published:** 2013-02-04

**Authors:** Fanrong Meng, Haiying Jia, Na Ling, Yinlei Xue, Hao Liu, Ketao Wang, Jun Yin, Yongchun Li

**Affiliations:** 1 College of Life Science, Henan Agricultural University, Zhengzhou, 450002, China; 2 National Engineering Research Centre for Wheat, Henan Agricultural University, Zhengzhou, 450002, China; 3 State Key Laboratory Cultivation Base of Crop Physiological Ecology and Genetic Improvement in Henan Province, Henan Agricultural University, Zhengzhou, 450002, China

**Keywords:** Argonaute, Cloning, Development, Gene expression, Wheat (*Triticum aestivum* L.)

## Abstract

**Background:**

Argonaute proteins are key components of RNA interference (RNAi), playing important roles in RNA-directed gene silencing. Various classes of Argonaute genes have been identified from plants and might be involved in developmental regulation. However, little is known about these genes in wheat (*Triticum aestivum*).

**Results:**

In this study, two full-length cDNAs of Argonaute were cloned from wheat, designated as *TaAGO1b* and *TaAGO4*. The cDNA of *TaAGO1b* is 3273 bp long and encodes 868 amino acids, with a predicted molecular weight of ~97.78 kDa and pI of 9.29. The 3157-bp *TaAGO4* encodes 916 amino acids, with a molecular mass of 102.10 kDa and pI of 9.12. Genomics analysis showed that *TaAGO1b* and *TaAGO4* contain 20 and 18 introns, respectively. Protein structural analysis demonstrated that typical PAZ and PIWI domains were found in both TaAGO1b and TaAGO4. From the highly conserved PIWI domains, we detected conserved Asp-Asp-His (DDH) motifs that function as a catalytic triad and have critical roles during the process of sequence-specific cleavage in the RNAi machinery. Structural modelling indicated that both TaAGOs can fold to a specific α/β structure. Moreover, the three aligned DDH residues are spatially close to each other at the “slicer” site of the PIWI domain. Expression analysis indicated that both genes are ubiquitously expressed in vegetative and reproductive organs, including the root, stem, leaf, anther, ovule, and seed. However, they are differentially expressed in germinating endosperm tissues. We were interested to learn that the two *TaAGOs* are also differentially expressed in developing wheat plants and that their expression patterns are variously affected by vernalization treatment. Further investigation revealed that they can be induced by cold accumulation during vernalization.

**Conclusions:**

Two putative wheat Argonaute genes, *TaAGO1b* and *TaAGO4*, were cloned. Phylogenetic analysis, prediction of conserved domains and catalytic motifs, and modelling of their protein structures suggested that they encode functional Argonaute proteins. Temporal and spatial expression analyses indicated that these genes are potentially involved in developmental regulation of wheat plants.

## Background

The RNA interference pathways are well-known for their critical roles in post-transcriptional gene silencing, and in triggering chromatin modifications [1]. Genetic, biochemical, and structural studies have implicated Argonaute (AGO) proteins as the catalytic core of the RNAi effector complex RISC; the first AGO gene was identified in *Arabidopsis thaliana*[2,3]. Plant AGO proteins are highly conserved basic proteins (approximately 100 kDa) with typical structural domains or amino acid motifs. These include a C-terminal PIWI (P-element induced wimpy testis) domain with an RNase-H-like fold, a central PAZ (Piwi Argonaute and Zwille) domain that binds small RNAs through the 3' end of the target RNA, and a MID domain, located between the PAZ and the PIWI domains, which anchors the 5' phosphate end of small RNAs onto Argonaute proteins [4,5]. Based on both their phylogenetic relationships and their capacity to bind to small RNAs, one can sort these proteins into three groups: I, which is the AGO-like subfamily (similar to AGO1 of *Arabidopsis thaliana*) that bind to microRNAs (miRNAs) and small interfering RNAs (siRNAs); II, the PIWI-like subfamily (closely related to the PIWI protein of *Drosophila melanogaster*), members of which bind to PIWI-interacting RNAs (piRNAs); and III, the WAGO subfamily (Worm-specific Argonautes) that bind to secondary siRNAs [5-7].

AGO-small-RNA complexes can repress the transcription of genes, target mRNAs for site-specific cleavage or general degradation, or block mRNA translation into protein. Some evidence suggests that Argonaute proteins play crucial roles in RNA-directed gene silencing, as well as being involved in the developmental regulation of plants [8-10]. Through genomic annotation, 10 and 18 AGO genes have been identified from *Arabidopsis thaliana* and rice (*Oryza sativa*), respectively [4,11]. Although some of these AGO members have been characterized, most remain unexplored in plants.

Wheat (*Triticum aestivum*) is a globally important crop, accounting for 20% of the calories consumed by humans [12]. Research that focuses on mechanisms for developmental regulation at the molecular level is very important for accelerating the progress of wheat improvement. AGO proteins, acting as key components in RNA-directed gene-silencing mechanisms, play significant roles in developmental regulation [9,10]. However, little is known about such genes in wheat. Here, we cloned and characterized two wheat AGO genes and analyzed their temporal and spatial expression patterns. We also investigated their potential involvement in development-specific gene regulation.

## Results

### Cloning of *TaAGOs* with full-length cDNA

We performed a TBLASTX analysis in the NCBI EST (expressed sequence tag) database ( http://www.ncbi.nlm.nih.gov/dbEST/) with two *Arabidopsis* Argonaute genes, *AGO1* [GenBank: NM_179453] and *AGO4* [GenBank: NM_128262]. Two groups of wheat ESTs proved to be highly homologous to *Arabidopsis* AGO1 and AGO4. Based on the conserved regions of their EST sequences, we designed specific primers (Additional file 1) for cloning the wheat Argonaute genes.

**Figure 1:**
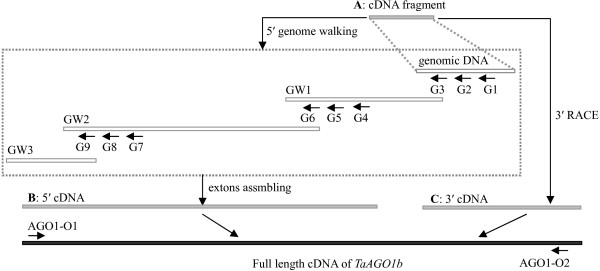
**Schematic diagram of *TaAGO1b *cloning. A**: cDNA fragment obtained from RT-PCR; **B**: assembled 5^′^-cDNA region based on genomic DNA; **C**: cDNA fragment acquired by 3^′^-RACE; GW1 – 3: DNA fragments obtained by genome-walking analysis

RT-PCR (reverse transcription-polymerase chain reaction) amplification was conducted with the primer combination of TaAGO1-1F and -1R. A 412-bp cDNA fragment (Figure 1-A) was cloned. To elongate the sequence of the wheat Argonaute gene, new primers were designed based on the cloned sequence and used in 5'- and 3'-RACE (Rapid Amplification of cDNA Ends). Although 5'-RACE analysis was performed several times, no satisfactory results were obtained. Therefore, we selected a genome-walking strategy to clone that 5' region. First, the genomic fragment corresponding to the cDNA region (Figure 1-A) was cloned and genome-walking primers (Additional file 1) were designed based on the sequence. Three rounds of genome-walking (Figure 1, GW1-3) were then conducted to obtain the 3958-bp genomic DNA. Finally, the 5'-cDNA region (Figure 1-B) was deduced by assembling exons ( http://genes.mit.edu/GENSCAN.html). The 3'-cDNA fragment (Figure 1-C) was analyzed by 3'-RACE. The full-length cDNA was obtained by assembling the three fragments indicated above (Figure 1-A-C), and the cDNA sequence between AGO1-O1 and -O2 was confirmed by RT-PCR cloning and sequencing. This wheat AGO gene (3273 bp long) encodes a putative protein of 868 amino acid residues, which is highly homologous to rice OsAGO1b [Swiss-Prot: Q7XSA2.3], and was designated as *TaAGO1b* [GenBank: JQ805149].

Using primers TaAGO4-1F and -1R, we cloned a 498-bp wheat cDNA fragment via RT-PCR amplification. Sequencing results showed that this cDNA is very similar to *Arabidopsis* AGO4. Based on the cloned sequence, we obtained its full-length cDNA by 5'- and 3'-RACE. Sequence analysis indicated that the cDNA from our wheat *AGO4* is 3157 bp long and encodes a putative protein of 916 amino acid residues. BLASTX analysis in NCBI revealed that it is highly homologous to *Arabidopsis* AGO4 [GenBank: AEC07929.1]. Thus, we designated it as *TaAGO4* [GenBank: JQ805150].

**Figure 2:**
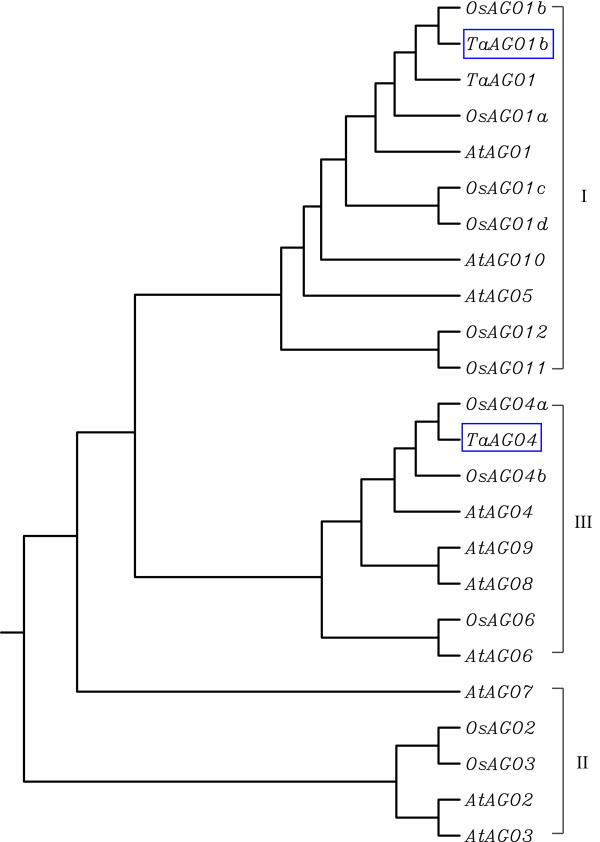
**Phylogenetic analysis of TaAGOs and other plant AGOs.** AtAGOs and OsAGOs are from *Arabidopsis* and rice, respectively. Three subfamilies are labeled at right margin. TaAGO1b and TaAGO4 are marked with boxes

Phylogenetic analyses of AGO plant proteins, including those from rice, *Arabidopsis*, and wheat, showed that TaAGO1b and TaAGO4 can be classified into two of the three groups described above (Figure 2). TaAGO1b, assigned to Group I, shares a high degree of homology with OsAGO1b, TaAGO1, OsAGO1a and AtAGO1. TaAGO4, as part of Group III, belongs to the same monophyletic subclass as rice OsAGO4a, OsAGO4b, and OsAGO6; and *Arabidopsis* AtAGO4, AtAGO6, AtAGO8, and AtAGO9. Three *Arabidopsis* AGOs (AtAGO2, 3, and 7) and two rice AGOs (OsAGO2 and 3) were sorted into Group II (Figure 2).

### Characteristics of TaAGOs

The sequence analysis at ExPaSy ( http://www.expasy.org) [13,14] indicated that TaAGO1b includes 868 amino acids, with a predicted molecular weight of ~97.78 kDa and theoretical pI of 9.29. TaAGO4 is 916 amino acids long and has a theoretical pI of 9.12 and a molecular mass of about 102.10 kDa.

**Figure 3:**
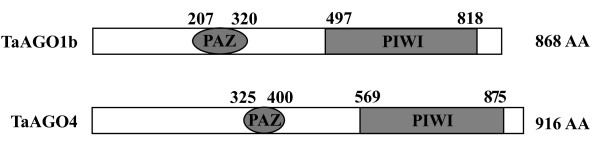
**Conserved domains and their location in TaAGOs.** PAZ and PIWI domains are represented by different shapes, and their locations are numbered according to amino acids

Both TaAGOs contain typical PAZ and PIWI conserved regions (Figure 3). The PAZ domain of TaAGO1b is composed of 114 amino acids (from Residue 207 – 320) and shares 82 to 95% homology with those of AGO1 proteins in other plant species. The PAZ domain of TaAGO4 comprises 76 amino acids (Residue 325 – 400) and has only limited homology with other plant AGO4 members. The C-terminal PIWI domain of TaAGO1b includes 322 amino acids (Residue 497 – 818), and has high similarity with that domain in other plant AGO1 members, e.g., OsAGO1b (97%), OsAGO1a (96%), and AtAGO1 (92%). The PIWI domain of TaAGO4 (Residue 569 – 875) shares 90%, 85%, and 77% homology with that of OsAGO4a, OsAGO4b, and AtAGO4, respectively. Generally, the PIWI domain displays a higher degree of similarity among plant Argonaute members, which supports a previous report of greater conservation of the PIWI domain but a poorly conserved PAZ domain [15].

**Figure 4:**
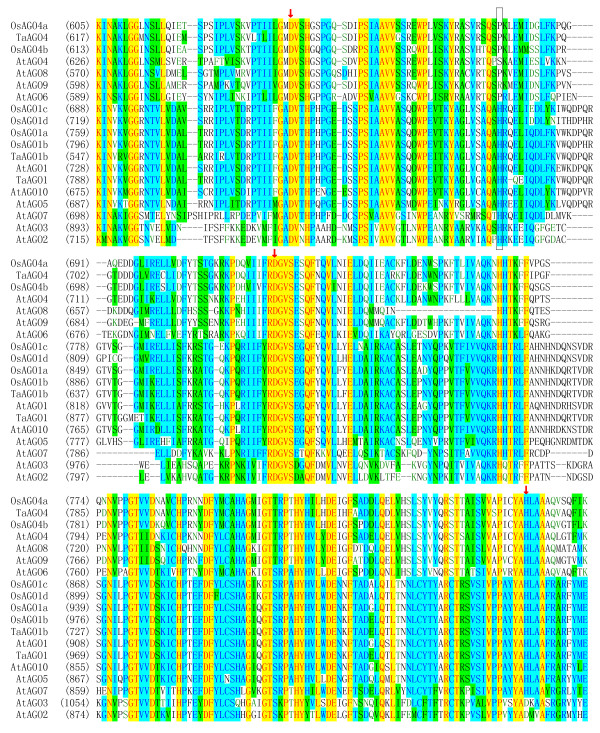
**Alignment of PIWI domains in plant AGOs.** Conserved Asp, Asp, and His (DDH) triad residues are marked with downward arrows. The H798 site of AtAGO1 is indicated by box. Amino acid positions for each PIWI domain are numbered

When we aligned the PIWI domains of plant AGOs, including TaAGOs and paralogs in rice and *Arabidopsis*, we detected a trio of conserved metal-chelating amino acids -- aspartate, aspartate, and histidine (DDH) -- in both TaAGO1b and TaAGO4 (Figure 4). These particular DDH residues play critical roles during the process of RNA-directed cleavage of target RNAs, acting as a catalytic triad [16]. Thus, the inclusion of a DDH triad in both wheat AGOs suggests that they are functionally similar to previously characterized AGOs in RNA-directed gene silencing. Previous research has shown that the conserved histidine at position 798 (H798) of AtAGO1 is essential for *in vitro* endonuclease activity [17]; this residue was also detected in TaAGO1b. By comparison, in TaAGO4, as well as in OsAGO4a, OsAGO4b, AtAGO6, and AtAGO8, the histidine at the 798th position was replaced by proline (P), whereas in AtAGO4 and AtAGO9, that site was switched to serine (S) and arginine (R), respectively (Figure 4).

**Figure 5:**
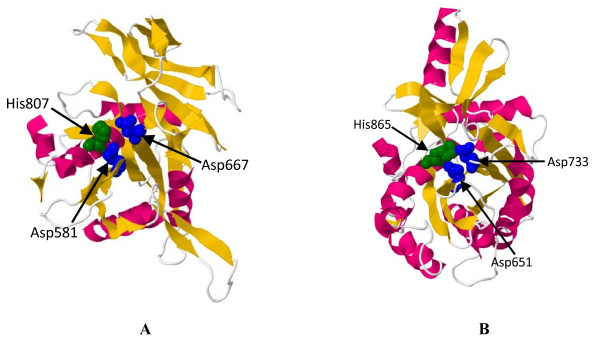
**Structural models of PIWI domains in TaAGO1b (A) and TaAGO4 (B).** DDH catalytic triad is colored blue for Asp (D) and green for His (H)

Structural modelling of the PIWI domain, with the Argonaute protein [PDB: 1u04] from *Pyrococcus furiosus* as template, indicated that TaAGOs can fold to a specific α/β structure that is dominated by a central mixed β-sheet and flanked by two long α-helices. Here, the three aligned DDH residues (Asp, Asp, and His) were spatially close to each other and located in the “slicer” site of the PIWI domain (Figure 5).

### Genomic sequence analysis of *TaAGO1b* and *TaAGO4*

**Figure 6:**
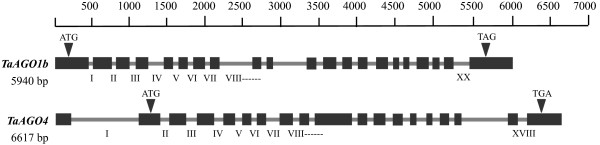
**Genomic characterization of *TaAGO1b *and *TaAGO4*.** Black boxes indicate exons; translational start and stop codons are shown

The genomic DNA sequence was analyzed by DNA amplification or genome-walking, using specific primers. *TaAGO1b* included 20 introns that varied in length from 72 to 449 bp. By contrast, 18 introns, 78 to 858 bp long, occurred in *TaAGO4* (Figure 6). The largest intron (858 bp) in *TaAGO4* was located in the region corresponding to the 5' UTR of mRNA.

### Spatial expression patterns of *TaAGOs*

**Figure 7:**
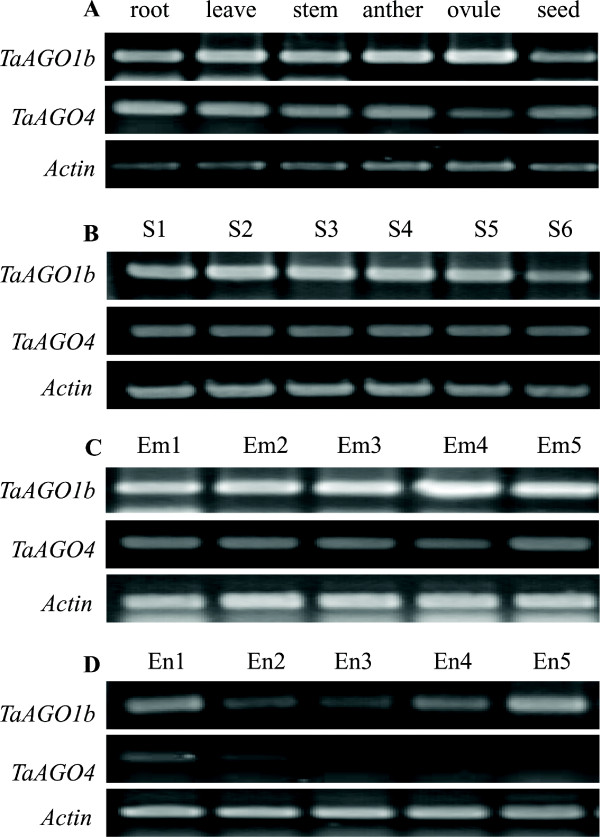
**Expression characteristics of two *TaAGOs *in different tissues.** (**A**) vegetative and reproductive organs. (**B**) Developing kernels; S1 – 5, represents 5, 10, 15, 20, and 30 days after pollination (DAP), respectively; S6, natural matured seed. (**C**) Embryo tissues of germinating seeds; Em1 – 5 represents 6, 12, 18, 24, and 48 h after imbibition, respectively. (**D**) Germinating endosperm tissues; En1 – 5 represents 6, 12, 18, 24, and 48 h after imbibition, respectively

Expression analysis via Semi RT-PCR demonstrated that both *TaAGO1b* and *TaAGO4* were highly expressed in all examined tissues, including the root, stem, leaf, anther, ovule, and mature seed (Figure 7-A), as well as in developing wheat kernels (Figure 7-B). During germination, *TaAGO1b* and *TaAGO4* were ubiquitously expressed in embryonic tissues (Figure 7-C) but only differentially expressed in the endosperm (Figure 7-D). The transcript level of *TaAGO4* in the endosperm tissues of germinating wheat seeds was greatly decreased.

### Changes in *TaAGO* expression in response to vernalization

**Figure 8:**
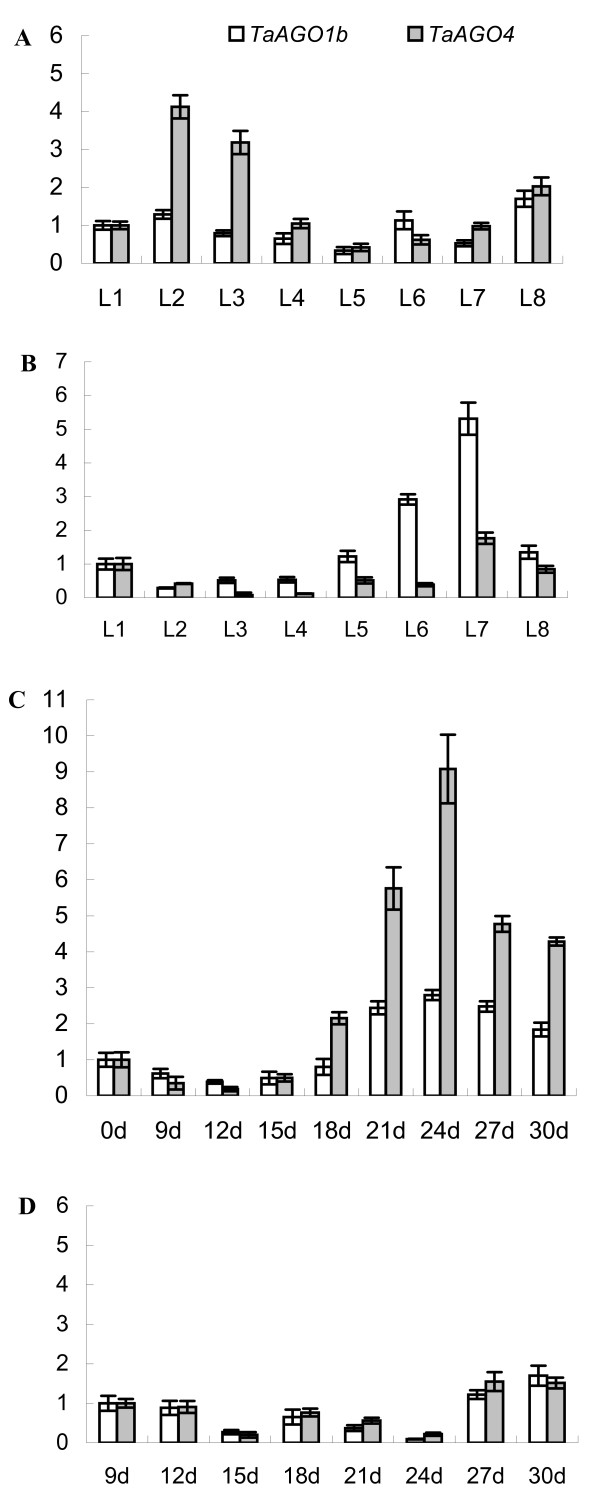
**Expression of *TaAGOs *during wheat plant development and vernalization.** (**A**) Wheat plants without vernalization treatment; L1 – 8, 1- to 8-leaf stages. (**B**) Wheat plants vernalized at germinating stage (exposed germinated seeds to 4°C for 30 d under darkness before planting). (**C**) Shoot tissues during vernalization treatment; 0 – 30 d, cold (4°C) treatment for 0 to 30 d, respectively. (**D**) Root tissues during vernalization

To investigate the expression patterns of *TaAGOs* over time, we harvested wheat leaves at the 1- to 8-leaf stages of development and performed quantitative real-time RT-PCR. Expression of *TaAGO4* was significantly up-regulated in the 2- and 3-leaf stages, while that of *TaAGO1b* was not obviously changed during our observation period (Figure 8-A). Vernalization treatment (exposing germinated seeds to 4°C for 30 d under darkness before planting) significantly affected their expression patterns, with *TaAGO4* no longer being up-regulated at the 2- and 3-leaf stages but *TaAGO1b* being significantly up-regulated at the 6- and 7-leaf stages (Figure 8-B).

For further examination of the effects of vernalization treatment, we monitored the expression of *TaAGOs* during cold accumulation. Both were induced in the shoot tissues, with expression of *TaAGO4* being greatly up-regulated at 18 to 30 d after cold treatment began but induction of *TaAGO1b* being relatively lower (Figure 8-C). However, no obvious induction of *TaAGOs* was detected in root tissues during the cold accumulation (Figure 8-D). These results indicated that both genes have important roles within the vernalization response, but their functioning might involve different regulatory mechanisms.

### *In silico* mapping of TaAGOs

From a set of wheat aneuploids and deletion stocks, over 16000 ESTs have been mapped in the wheat chromosome / chromosome bins [18]. Those results are very useful for *in silico* mapping analysis. Through BLASTN searches, we identified mapped ESTs that are homologous to wheat AGO genes (Additional file 2). Based on sequence similarities and positions, we were able to map *TAGO1* to the long arm of 7D and *TaAGO4* to the short arm of 3A, 3B, and 3D on the wheat chromosomes.

## Discussion

Regulation of gene expression at the post-transcriptional level plays a critical role in plant development. RNAi pathways are involved in post-transcriptional gene silencing and chromatin modifications. Argonaute proteins comprise a highly conserved family whose members have been implicated in RNAi- and related pathways in plants [15]. We isolated two wheat AGO genes with full-length cDNA, *TaAGO1b* and *TaAGO4*. Both TaAGOs are basic proteins (pI ~ 9.0) with predicted molecular weights of ~100 kDa that are similar to other AGO members in eukaryotes [6,19]. Argonautes are characterized by two highly conserved domains, PAZ and PIWI, that have important roles during the processes of RNAi-mediated gene regulation [4]. The PAZ domain is a key region that interacts with the 3' end of small RNAs and determines the RNA-binding specificity of Argonautes. The PIWI domain binds to the 5' end and also interacts with the target RNA [5]. Among rice AGOs, homology of PAZ domains is relatively lower (~21%), whereas the PIWI domains are highly conserved and show homologies of >90% [16]. We also found that the PIWI domain of AGOs is highly conserved among *Arabidopsis*, rice, and wheat. That domain exhibits extensive homology to RNase H; some Argonaute proteins cleave the target RNAs that have sequences complementary to the small RNAs [17]. During this process of sequence-specific cleavage, three conserved metal-chelating residues in the PIWI domain play critical roles and function as a catalytic triad [16,20]. In addition, PIWI proteins with degenerate active catalytic motifs of ‘D-D-H/D/E/K’ show deduced slicer or slicer-like activity [6]. We detected this conserved DDH triad in both TaAGO1b and TaAGO4. Structural modelling indicated that the DDH residues were spatially close to each other and located at the “slicer” site of the PIWI domains. These features demonstrate the regulatory functioning of TaAGOs within the RNAi machinery.

In plants, RNAi-dependent pathways have been widely implicated in the control of gene expression and involvement in various developmental events [21,22]. Components of the RNAi machinery participate in the maintenance of undifferentiated cells in the shoot apical meristem (SAM), initiation of lateral organ primordia from SAM and floral meristems, and the formation of male and female germ cells [11,23,24]. In *Arabidopsis*, *AGO1* and *AGO10* regulate the termination of floral stem cells through two microRNAs, miR172 and miR165/166 [10]. In rice, microarray-based expression profiling has shown that the counterparts of *Arabidopsis AGO1* and *AGO4* are significantly up-regulated at the onset of floral development [16]. In our study, *TaAGO1b* and *TaAGO4* were ubiquitously expressed in both vegetative and reproductive organs, but differentially expressed in germinating endosperm tissues. Expression of *TaAGO4* was immediately and greatly decreased in those endosperm tissues after seed imbibition, suggesting that TaAGO4 is involved in the RNA-directed silencing of certain target genes in the endosperm (such as those that encode amylases). Moreover, this down-regulation of TaAGO4 was consistent with the up-regulation of enzymes during germination, which are necessary for breaking down endosperm starch into sugars to nourish the growing seedlings.

We were also interested to find that *TaAGO4* was significantly up-regulated in leaves at the 2- and 3-leaf stages, and that this expression pattern was also significantly affected by vernalization treatment. However, neither *TaAGO1b* nor *TaAGO4* was induced by treatment at 4°C for 15 d. Nevertheless, after 21 d of cold accumulation, both were significantly up-regulated. This suggested that *TaAGO1b* and *TaAGO4* are involved in the RNA-directed silencing of certain flowering repressors in shoots during wheat vernalization. In root tissues, no obvious induction of *TaAGOs* was detected under cold stress, a finding consistent with previous reports that *TaAGO1* (a homolog of *TaAGO1b*) is not responsive to low-temperature stress, but is significantly regulated by dehydration [25]. Thus, our results indicate potential involvement by *TaAGO1b* and *TaAGO4* in development-specific gene regulatory mechanisms. This provides insight into probable functions for TaAGOs and serves as a new starting point for further investigations aimed at understanding RNAi-dependent gene regulation during seed germination and vernalization in wheat.

## Conclusions

Argonaute proteins are key components of RNA-directed gene silencing, playing important roles in the regulation of plant development. We cloned and characterized two Argonaute genes from wheat, *TaAGO1b* and *TaAGO4*. Our results suggested they encode functional AGO proteins. Interestingly, these two genes manifested different patterns of expression during seed germination and plant growth, as well as in response to vernalization treatment. This demonstrates that *TaAGO1b* and *TaAGO4* are probably involved in the developmental regulation of wheat plants.

## Methods

### Plant sample preparation

The roots, stems, leaves, anthers, ovules, and mature seeds were sampled from a winter wheat cultivar, ‘Jing 841’. Developing seeds were collected at 5, 10, 15, 20, and 30 DAP from field-grown plants. For germination treatment, mature seeds were surface-sterilized in 5% sodium hypochloride for 15 min and washed four times (2 min each) with sterilized water. They then imbibed water from moist filter paper in Petri dishes in a temperature-controlled cultivation chamber (16-h photoperiod at 25°C). Embryo and endosperm tissues were isolated at 6, 12, 24, 48, and 72 h after imbibition.

To monitor the expression of wheat AGOs over time, we placed seeds in pots and cultured them in a temperature-controlled chamber at 25°C and under a 16-h photoperiod. Leaves were harvested at the 1- to 8-leaf stages.

For studying the effects of vernalization treatment on AGO expression, seeds imbibed water for 24 h and were then transferred for 30 d to a cold chamber (4°C in the dark). After vernalization, they were planted in pots and cultured in a temperature-controlled chamber at 25°C and under a 16-h photoperiod. Leaves were harvested at the 1- to 8-leaf stages. To examine the expression patterns of AGOs during this vernalization treatment, we sampled shoot and root tissues on Days 0, 9, 12, 15, 18, 21, 24, 27, and 30. All materials were prepared from three replicates of each independent treatment.

### Preparation of total RNA and genomic DNA

Total RNA was extracted as previously described [26], and the integrity of those samples was assessed by agarose gel (0.8%) electrophoresis. The concentration and purity of RNA were determined from the A260/A280 ratio, using a UC800 nucleic acid-protein analyzer (Beckman Co., USA). Genomic DNA was extracted from the wheat leaves according to the CTAB (hexadecyltrimethylammonium bromide) method [27].

### Cloning of cDNAs

Two *Arabidopsis* Argonaute genes, *AGO1* [GenBank: NM_179453] and *AGO4* [GenBank: NM_128262], were used in our TBLASTX search against the EST database. From this, we identified two groups of wheat EST sequences -- GenBank CF133307, CA741561, CK155094, CJ900837, CA726349, and BE586111; and GenBank BT009411, BQ841772, HX190168, CB307539, BJ244578, and CJ679989, which are highly homologous to *AGO1* and *AGO4*, respectively. Based on the conserved regions in each of the wheat groups, we designed primer pairs TaAGO1-1F/1R and TaAGO4-1F/1R (Additional file 1) for use in cDNA cloning of the wheat genes. RT-PCR was conducted with 2 μl of cDNA as template plus 125 pmol of each primer in a 20-μl reaction system that contained 0.2 mM of each dNTP, 1.5 mM MgCl_2_, and 1 U of *Taq* polymerase. The PCR program was as follows: 5 min at 94°C; then 40 cycles of 1 min at 95°C, 1 min at 53°C, and 1 min at 72°C; plus a final extension of 10 min at 72°C. After the products were separated by 1% agarose gel electrophoresis, their bands were recovered and sub-cloned into the pBS-T vector (TakaRa, China) for sequencing. The RACE technique was performed with a TaKaRa kit (5^′^-Full RACE Kit and 3^′^-Full RACE Core Set Ver. 2.0) according to the manufacturer’s instructions. Specific primers are listed in Additional file 1. The gene-specific primer GP1 was combined with the outer primer of 3^′^-RACE, while GP3 was combined with that of 5^′^-RACE. Specific primers GP2 and GP4 were combined with 3^′^- and 5^′^- RACE inner primers, respectively.

### Confirmation of full-length cDNAs

To verify the assembled sequences, we designed gene-specific primers and sequenced the RT-PCR products. Primers TaAGO1-O1 and TaAGO1-O2 were used to clone TaAGO1b, while TaAGO4-O1 and TaAGO4-O2 were used for TaAGO4 amplification (Additional file 1).

### Genomic characterization of *TaAGOs*

We used specific primers in genomic DNA amplification to analyze the genomic sequence of *TaAGO1b*. Primers TaAGO1-1F/R and TaAGO1-2F/R were applied in cloning the 3´-region whereas the 5´-region was amplified with a genome-walking kit (Takara, Japan). Those genome-walking primers (TaAGO1-G1 through TaAGO1-G9) are listed in Additional file 1 and their locations are shown in Figure 1. Six pairs of gene-specific primers (AGO4-1F/R through AGO4-6F/R) were used for genomic DNA amplification of TaAGO4.

### Gene sequence analysis and *in silico* mapping

A BLAST search was performed at NCBI. The amino acid sequence was deduced and its structure predicted at ExPaSy [13,14]. ClustalW ( http://www.ebi.ac.uk/clustalw/) was used for alignment and phylogenetic analysis. A structural model of the PIWI domain was constructed with the HHpred server, using Modeller 8.0 [28]. The selected template structure (i.e., the best hit from HHpred) was that of the *P*. *furiosus* Argonaute protein (PfAGO; UniProt code: Q8U3D2, PDB id 1U04) [19]. The model of the PIWI domain included Residues 825 to 1070. *In silico* mapping on the Chinese Spring deletion map was conducted at Graingenes ( http://wheat.pw.usda.gov/GG2/blast.shtml).

### Gene expression analysis

Gene-specific primer pairs AGO1-F/R and AGO4-1F/R were used for RT-PCR analysis, and the actin gene [GenBank: AB181991] served as the endogenous control. Primers included TaAc-F: GTTCCAATCTATGAGGGATACACGC and TaAc-R: GAACCTCCACTGAGAACAACATTACC. RT-PCR was carried out with 2 μl of cDNA as template and 125 pmol of each primer in a 20-μl reaction system that contained 0.2 mM of each dNTP, 1.5 mM MgCl_2_, and 1 U of *Taq* polymerase. The PCR program was as follows: 5 min at 94°C; then 28 cycles of 1 min at 95°C, 1 min at 53°C, and 1 min at 72°C; plus a final extension of 10 min at 72°C. The products were separated by 1% agarose gel electrophoresis. Real-time quantitative RT-PCR was performed as previously described [26], with the ubiquitin gene (GenBank Accession No: AY297059) used as the endogenous control. Primers included UF: ATCCAGGACAAGGAGGGCA and UR: CGGAGACGGAGCACCAAG. All other primers are listed in Additional file 1.

## Abbreviations

AGO: Argonaute; CTAB: Hexadecyltrimethylammonium bromide; DAP: Days after pollination; EST: Expressed sequence tag; PAZ: Piwi Argonaute and Zwille; PIWI: P-element induced wimpy testis; RACE: Rapid Amplification of cDNA Ends; RNAi: RNA interference; RT-PCR: Reverse transcription-polymerase chain reaction

## Competing interests

The authors declare that they have no competing interests.

## Authors’ contributions

FM performed the bioinformatics, gene-cloning, and phylogenetic analyses. HJ, NL, YX, and HL were involved in the gene-cloning and expression assays. KW participated in the bioinformatics analysis. FM, JY, and YL designed and prepared the manuscript. All authors read and approved the final manuscript.

## Supplementary Material

Additional file 1 **Primers for cloning and expression analyses.** Listed all of primers used in the study. Click here for file

Additional file 2 ***In silico *mapping of *TaAGOs.*** Listed the wheat ESTs homologous to *TaAGO1b* and *TaAGO4* and their locations in wheat chromosomes. Click here for file
