# Supplementary material for: Cloning and characterization of two Argonaute genes in wheat (Triticum aestivum L.)
Source: BMC Plant Biol. 2013 Feb 4;13:18. doi: 10.1186/1471-2229-13-18 (PMC3621544; doi:10.1186/1471-2229-13-18)
Supplement: Additional file 1 — Primers for cloning and expression analyses. Listed all of primers used in the study. [file 1471-2229-13-18-S1.doc]

Additional file 1. Primers for cloning and expression analyses

| Primer | sequnence (5´-3´) | Function |
| --- | --- | --- |
| TaAGO1-1F | GATTGGTATCCCAGTGTTGTCTTA | GC, EA, GA |
| TaAGO1-1R | GGTTTCTGTCCAGTTGCCCTC | GC, EA, GA |
| TaAGO1-2F | AGTGAGTGCCCAAGCCCATA | GA |
| TaAGO1-2R | TGCTTCAAGTAGCCTCACGGT | GA |
| TaAGO1-G1 | TCTTCTCCAGGGTGAGGATGTGT | GW |
| TaAGO1-G2 | TCTGTCGGTAACAAGGCGAATCC | GW |
| TaAGO1-G3 | GAATGGTTGGAGGTCAGTGAAGTA | GW |
| TaAGO1-G4 | CCGACTCTACCACCATTGACCAT | GW |
| TaAGO1-G5 | AGTGAGAAAGAAGGACAGCAGCAA | GW |
| TaAGO1-G6 | TGTGTGATTCGTTGCCACTTCTA | GW |
| TaAGO1-G7 | TGGCAGTAGGCAATTCCCGT | GW |
| TaAGO1-G8 | TTCCCGTAGCACAATGTCAAGC | GW |
| TaAGO1-G9 | GTCCTCGAAGTAAATGGTAATGGC | GW |
| TaAGO1-GP1 | TGTTACCGACAGACCGACCATA | RA3 |
| TaAGO1-GP2 | CCAAGTACGCAGGATTAGTGAGTG | RA3 |
| TaAGO1-GP3 | TTTCTGTCCAGTTGCCCTCT | RA5 |
| TaAGO1-GP4 | TTATGGTCGGTCTGTCGGTA | RA5 |
| TaAGO1-O1 | GAATGGGTAAATGTGGGGATAG | GC |
| TaAGO1-O2 | GTTGAACAGCATCACCAATACTAATA | GC |
| TaAGO1-qF | ATGCTTTGGCAAGGAGG | qEA |
| TaAGO1-qR | TATGGGCTTGGGCACTC | qEA |
| TaAGO4-1F | CCAGAAGGTTGACCACCCTAATA | GC, EA, GA |
| TaAGO4-1R | TGTGTAAAGCCCTGAGCTATCG | GC, EA, GA |
| TaAGO4-2F | AAGAAGCCATTCGGGTTATT | GA |
| TaAGO4-2R | ATGCCAATATGCTCACTTACATC | GA |
| TaAGO4-3F | TTGGCAGGTACTGAAACGC | GA |
| TaAGO4-3R | ATTGTCAGGAGATCCAGGTATG | GA |
| TaAGO4-4F | CTACTTGCAAATGTCAGTGGTC | GA |
| TaAGO4-4R | CAGCAACCAGCTTAGGAACAC | GA |
| TaAGO4-5F | CCCTGATCGCTCGTCCTG | GA |
| TaAGO5-5R | AAAGTGACTGGCGGACTAACAG | GA |
| TaAGO4-6F | GATCAGTTCAGCGGTTCG | GA |
| TaAGO5-6R | CAGCAATAGACAATACTGGACC | GA |
| TaAGO4-GP1 | TGTGAAGAACCGTGGCATTG | RA3 |
| TaAGO4-GP2 | TTGTTGAGAAGTCCAGGCAGAAGC | RA3 |
| TaAGO4-GP3 | GGCTTCTGCCTGGACTTCTCAA | RA5 |
| TaAGO4-GP4 | CTCAATGCCACGGTTCTTCACA | RA5 |
| TaAGO4-O1 | CAACGGCAAGGCTTCCTCC | GC |
| TaAGO4-O2 | GGACTTTCTCATGGGGACGAG | GC |
| TaAGO4-qF | CAAAGGAGCACGACAGC | qEA |
| TaAGO4-qR | GCAACCAGCTTAGGAACAC | qEA |

GC: gene cloning; EA: expression analysis; GA: genomic analysis; GW: genome walking; RA3: 3' -RACE; RA5: 5'-RACE; qEA: real-time q-RT-PCR expression analysis.
