# Supplementary material for: Cloning and characterization of two Argonaute genes in wheat (Triticum aestivum L.)
Source: BMC Plant Biol. 2013 Feb 4;13:18. doi: 10.1186/1471-2229-13-18 (PMC3621544; doi:10.1186/1471-2229-13-18)
Supplement: Additional file 2 — In silico mapping of TaAGOs. Listed the wheat ESTs homologous to TaAGO1b and TaAGO4 and their locations in wheat chromosomes. [file 1471-2229-13-18-S2.doc]

Additional file 2. *In silico* mapping of *TaAGOs*

| Gene name | Homology EST accession | Chromosome | Bin location |
| --- | --- | --- | --- |
| *TaAGO1* | BG312663 | 7DL | 7DL3-0.82-1.00 |
| *TaAGO4* | BE591466 | 3AS  3BS  3DS | C-3AS2-0.23  C-3BS1-0.33  3DS3-0.24-0.55 |
